# Uniparental disomy screen of Irish rare disorder cohort unmasks homozygous variants of clinical significance in the *TMCO1* and *PRKRA* genes

**DOI:** 10.3389/fgene.2022.945296

**Published:** 2022-09-14

**Authors:** B. Molloy, E. R. Jones, N. D. Linhares, P. G. Buckley, T. R. Leahy, B. Lynch, I. Knerr, M. D. King, K. M. Gorman

**Affiliations:** ^1^ Genuity Science, Dublin, Ireland; ^2^ Department of Paediatric Immunology, Children’s Health Ireland at Crumlin, Dublin, Ireland; ^3^ Department of Paediatrics, Trinity College, University of Dublin, Dublin, Ireland; ^4^ Department of Paediatric Neurology and Clinical Neurophysiology, Children’s Health Ireland at Temple Street, Dublin, Ireland; ^5^ School of Medicine and Medical Sciences, University College Dublin, Dublin, Ireland; ^6^ National Centre for Inherited Metabolic Disorders, Children’s Health Ireland at Temple Street, Dublin, Ireland

**Keywords:** genetic screen, rare disease, genomics, uniparental, pathogenic

## Abstract

A uniparental disomy (UPD) screen using whole genome sequencing (WGS) data from 164 trios with rare disorders in the Irish population was performed to identify large runs of homozygosity of uniparental origin that may harbour deleterious recessive variants. Three instances of whole chromosome uniparental isodisomy (UPiD) were identified: one case of maternal isodisomy of chromosome 1 and two cases of paternal isodisomy of chromosome 2. We identified deleterious homozygous variants on isodisomic chromosomes in two probands: a novel p (Glu59ValfsTer20) variant in *TMCO1*, and a p (Pro222Leu) variant in *PRKRA*, respectively. The overall prevalence of whole chromosome UPiD in our cohort was 1 in 55 births, compared to 1 in ∼7,500 births in the general population, suggesting a higher frequency of UPiD in rare disease cohorts. As a distinct mechanism underlying homozygosity compared to biallelic inheritance, the identification of UPiD has important implications for family planning and cascade testing. Our study demonstrates that UPD screening may improve diagnostic yields by prioritising UPiD chromosomes during WGS analysis.

## Introduction

Uniparental disomy (UPD) is generally a consequence of meiotic nondisjunction, where one or more chromosome pairs are of uniparental origin (Engel, 1980; Yamazawa et al., 2010; Del Gaudio et al., 2020). Nondisjunction events in meiosis I and II can lead to heterodisomy (UPhD), where both homologs of a chromosome are inherited from one parent, or isodisomy (UPiD), where two identical copies of one homolog are inherited from a single parent, respectively. This inheritance error may affect part or the whole chromosome.

A previous study on 214,915 trios estimated the prevalence of UPD (UPhD and UPiD) in the general population at 1 in ∼2,000 births, with unique per-chromosome rates that reflect the susceptibility of individual chromosomes to meiotic nondisjunction (Nakka et al., 2019). Independent assessments in clinical cohorts with diverse phenotypic indications, estimate the incidence of UPD to be between 1 in 176–500 births (King D. A. et al., 2014; Yauy et al., 2020; Scuffins et al., 2021). Whole chromosome UPiD was identified as the least common subtype of UPD, accounting for 27% of all uniparental disomy cases in 916,712 parent-child duos from the 23 and Me dataset, compared to 37% UPhD and 36% partial UPiD. The derived prevalence of UPiD is estimated to be 1 in ∼7,500 in the general population (Nakka et al., 2019).

Heterozygous deleterious variants on an affected chromosome in one parent may become homozygous in offspring as a consequence of partial or complete UPiD. Whole-genome sequencing (WGS) data is a powerful resource for investigating UPD inheritance errors in patients with suspected rare Mendelian disorders (Bis et al., 2017).

Here, we describe an identity-by-descent UPD screen of WGS data from 164 parent-child trios with rare disorders. Rare variant analysis was performed to identify deleterious variants on affected chromosomes. We show that applying *in silico* methods for the screening of UPD events in every patient with a suspected Mendelian disorder focuses WGS analysis and can improve diagnostic yields.

## Methods

### Samples and DNA isolation

The Research Ethics Committee of Children’s Health Ireland (CHI) at Temple Street approved the study protocol (Study number: 16.032). Informed consent was obtained according to current ethical and legal guidelines. Probands with undiagnosed rare diseases and both parents were recruited in CHI at Temple Street. The probands had a wide variety of indications for genetic testing. Genomic DNA was isolated at Genuity Science, Ireland, from whole peripheral blood using Qiagen’s Flexigene precipitation chemistry on FlexSTAR PLUS automated isolation instrument (AutoGen Inc., Holliston, MA, United States), according to the manufacturer’s protocol.

### Whole-genome sequencing (WGS)

Whole-genome sequencing was performed using Illumina TruSEQ DNA PCR-free whole-genome sequencing with 2 × 150 bp pair-end sequencing reads on a NovaSeq-6000 (Illumina Inc, San Diego, CA, United States), following the manufacturer’s protocol. The minimum mean sequencing coverage was 30x, with a minimum of 95% of target bases covered at least ten times. Using an automated Sentieon (Freed et al., 2017) based pipeline, raw sequencing FASTQ data were aligned to the GRCh38/hg38 reference genome build *via* Burrows-Wheeler aligner (BWA) and variants were called using mathematical models of the Best Practices Genome Analysis Toolkit (GATK).

### UPD screen

For each trio, GVCF files containing WGS data were merged and joint genotyped using GATK (Poplin et al., 2017). Biallelic variants with a minor allele frequency of ≥ 0.4 in Non-Finnish Europeans from the GnomAD dataset (Karczewski et al., 2020) were called in each trio using BCFtools. Genotypes were filtered for a minimum depth of 15, a maximum depth of 100 and a minimum genotype quality of 15 using VCFtools. Only single nucleotide polymorphisms were analysed. Approximate regional coverage was determined using the DP and MIN_DP fields in GVCF files. UPD across each chromosome was assessed by calculating the percentage of AB genotypes in the proband where one parent had an AA genotype, and the other parent had a BB genotype. This was compared to chromosome-level heterozygosity (calculated as the percentage of heterozygous sites across all non-missing sites) for all probands in the dataset (Supplementary Figure S1). Whole chromosome UPiD was identified in the case of extreme outliers in each of the two metrics (>90% uniparental skewing of genotypes and <10% of heterozygosity across the chromosome). Whole chromosome UPhD was defined as >90% uniparental skewing with >30% heterozygosity across the chromosome. To screen for partial UPD, each chromosome was divided into 5 Mb overlapping windows where each window started at a genotype which exhibited Mendelian error. Windows with 50 Mendelian errors were found. In each of these windows, all positions where the parents were homozygous for different alleles were examined. Windows where the proband exhibited >10% heterozygosity at these loci were removed. Overlapping windows were merged and the average coverage across these regions was calculated using a custom R script. The regional coverage was compared to the average genome-wide coverage which was calculated using the Sentieon implementation of the Picard CollectWgsMetrics algorithm (Freed et al., 2017). To distinguish between possible deletions and UPD, only regions with >90% of the average genome-wide coverage were considered candidate regions for partial UPD. For male probands (*n* = 95), only autosomes were analysed; for female probands (*n* = 69) autosomes and the X chromosome were examined. Direction of UPD was assessed by calculating the percentage of genotypes in the proband that were consistent with inheritance from either parent, at sites where the parents were homozygous for different alleles. We did not screen for mosaic disomy events.

### WGS rare variant analysis

VCF and BAM files from the trios were uploaded to Genuity Science Clinical Sequence Analyzer (CSA^®^). All data was stored according to genomic position in the Genuity Science Genomically Ordered Relational Database (GORdb) to facilitate rapid access by the CSA^®^ (version 4.18) user interface and Sequence Miner visualisation software, which were used for variant analysis. Variants were annotated for functional effect by Variant Effect Prediction (VEP-Ensembl, Version 96.2) and annotated with allele frequencies from publicly available large population databases such as Single Nucleotide Polymorphism database (dbSNP) and Genome Aggregation Database (GnomAD) (Karczewski et al., 2020), as well as an internal database of Irish controls. The analysis was limited to a subset of genes based on case-specific HPO terms, gene panels and OMIM classifications. Only rare variants (Minor Allele Frequency <0.03) classified as moderate or high impact by VEP were analysed. Alamut Visual software (version 2.12, Interactive Biosoftware) was used to analyse the impact of candidate variants.

### Sanger sequencing

Sanger sequencing was performed using the BigDye^®^ Direct Cycle Sequencing Kit in combination with the BigDye XTerminator™ Purification Kit (Applied Biosystems, Foster City, CA, EUA) and the Applied Biosystems SeqStudio™ Genetic Analyzer. Sequencing data was analysed using the software GeneStudio™ Professional Edition Version 2.2.0.0 (GeneStudio, Inc.).

## Results

**FIGURE 1:**
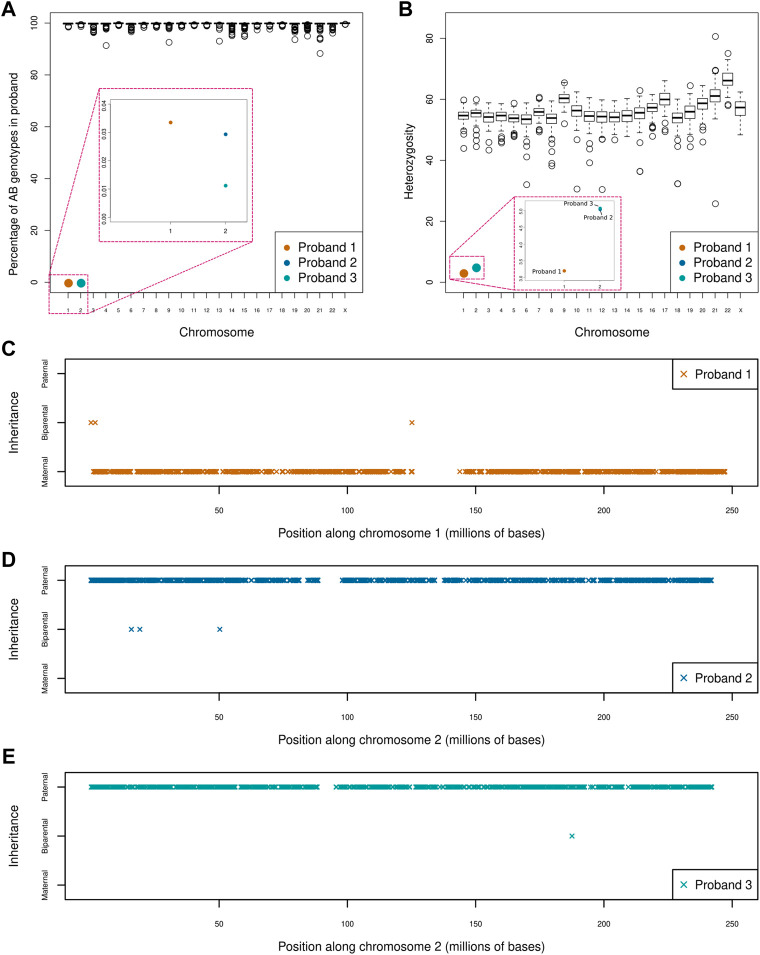
Uniparental disomy screen of 164 parent-child trios **(A)** Boxplot shows the percentage of AB genotypes in each child (represented by circles) when one parent has an AA genotype and the other parent has a BB genotype across each chromosome. The boxes in red indicate a zoom of that region of the plot for ease of visualisation. Three clear outliers can be seen affecting chromosome 1 (P1) and chromosome 2 (P2 and P3). Large regions of apparent Mendelian error across single chromosomes (e.g., in chromosomes 4, 9, and 14) were identified. An assessment of coverage across these regions confirmed these events to be a consequence of inherited or *de novo* deletions rather than partial UPD events. One of the large deletions had previously been identified by chromosomal microarray **(B)** Boxplot shows the percentage heterozygosity across each chromosome for every proband **(C–E)** Shows the direction and extent of disomy in P1 **(C)**, P2 **(D)** and P3 **(E)**. Proband 1 shows maternal isodisomy of chromosome 1 with 99.97% of genotypes matching that of the mother, while proband 2 and 3 exhibits paternal isodisomy of chromosome 2 with 99.99 and 99.97% of genotypes matching those of the father, respectively. This indicates that all three UPiD events affect the whole chromosome (these small deviations from 100% were attributed to sequencing and genotype calling errors). Only genotypes where the parents were homozygous for different alleles were used in this analysis.

Our UPD screen of 164 trios revealed three instances of whole chromosome UPiD: one case of maternal isodisomy of chromosome 1 (P1) and two cases of paternal isodisomy of chromosome 2 (P2 and P3). Uniparental disomy was detected by isolating large chromosomal regions where homozygous genotypes in the proband could not be ascribed to biparental inheritance (Figure 1A). No partial UPD events were found in our dataset (with a 5 Mb limit of resolution). Heterozygosity across each chromosome was assessed to differentiate between UPiD and UPhD (Figure 1B). Heterozygosity across chromosome 1 (P1) and chromosome 2 (P2 and P3) approached zero, indicative of UPiD. Some other probands exhibited low heterozygosity across their chromosomes which was due to recent consanguinity in their family tree. Conversely, one proband exhibited high levels of heterozygosity on chromosome 21. This proband had Down Syndrome.

Analysis of WGS data from all three probands prioritised rare homozygous variants in the isodisomic chromosomes which were not homozygous in the parents’ samples. Only variants in genes associated with the probands’ phenotypes were analysed (see phenotype summary on Table 1 and detailed clinical history in Supplementary Data). A homozygous frameshift variant at position chr1:165,759,558 c.176_177del p. (Glu59ValfsTer20) (NM_019026.6) in the Transmembrane and coiled-coil domains protein 1 (*TMCO1*) gene (MIM: 614123) was identified in P1. The genome of P1 was sequenced to a mean coverage of 30.35x. The genotype position had 0x coverage while the position 3’ of the deletion on the forward strand had 25x coverage and a genotype call ratio of 1. The variant is absent from gnomAD (Karczewski et al., 2020). Bi-allelic loss-of-function variants in *TMCO1* are associated with Craniofacial dysmorphism, skeletal anomalies, and intellectual disability syndrome (CFSMR; MIM: 213980). The clinical description of P1 is consistent with the spectrum of the condition (Table 1; Supplementary Data).

**TABLE 1:** Summary of clinical features and analysis results. Phenotype column shows HPO terms used in analysis.

Proband	UPiD	HGVS	ACMG	HPO terms
P1	Chr 1, maternal	NM_019026.6 (*TMCO1*): c.176_177del p. (Glu59ValfsTer20)	Pathogenic	Broad thumb, adducted thumb, high palate, triangular mouth, hypertelorism, axial hypotonia, scoliosis, Sprengel anomaly, hemivertebrae, bicuspid aortic valve, moderate intellectual disability, hand tremor
P2	Chr 2, paternal	NM_003690.5(*PRKRA*): c.665C>T p. (Pro222Leu)	Likely pathogenic	Abnormality of mitochondrial metabolism, easy fatigability, dysarthria, ataxia, encephalopathy
P3	Chr 2, paternal	N/A	N/A	Lymphopenia, specific pneumococcal antibody deficiency
HPO terms used in analysis and does not describe all clinical features. Please see **Supplementary Data** for detailed summary				

Analysis of P2 identified a previously reported homozygous missense variant; chr2:178436264G>A c.665C>T p. (Pro222Leu) (NM_003690.5) in the Protein kinase, interferon-inducible double-stranded RNA-dependent activator (*PRKRA*) gene. The genome of P2 was sequenced to a mean coverage of 41.79x. The genotype position had 48x coverage and a genotype call ratio of 1. The p. (Pro222Leu) missense variant is a known variant associated with a dystonia-parkinsonism phenotype (MIM: 612067). The variant allele frequency is 0.00009905 on gnomAD v2.1.1, with no homozygotes reported. It has been reported in dbSNP (rs121434410) and classified as pathogenic in ClinVar (VCV000006346.4).

No homozygous pathogenic variants were identified in P3. The variants identified in P1 and P2 were confirmed by Sanger sequencing. A review of P1 and P2 at a multidisciplinary meeting confirmed clinical correlation with findings. Both variants were classified according to ACMG guidelines (Richards et al., 2015) (Table 1).

## Discussion

We applied a WGS screening approach to identify UPD events in 164 probands from an undiagnosed cohort. Chromosomes with whole chromosome isodisomy were prioritised in the WGS analysis for deleterious variation. We identified three independent events, two of which rendered parental heterozygous pathogenic variants homozygous in probands (P1 and P2). The maternal UPiD event on chromosome 1 unmasked a novel frameshift variant in the *TMCO1* gene, predicted to produce two null allele, associated with CFSMR (MIM:213980). The paternal UPiD event on chromosome 2 in P2 uncovered a recurrent missense variant in the *PRKRA* gene reported in Dystonia 16 (MIM: 612067).

Recent studies provide estimates on the incidence of UPD in clinical cohorts with diverse phenotypic indications, ranging from 1 in 176–500 (King D. A. et al., 2014; Yauy et al., 2020; Scuffins et al., 2021), compared to 1 in 2,000 in the general population (Nakka et al., 2019) One study found that approximately half of UPD events were considered diagnostic or indirectly diagnostic (Scuffins et al., 2021). Clinically significant homozygosity as a consequence of UPD accounted for 1699 diagnoses, six of which localised to chromosome 1 (Scuffins et al., 2021). The incidence of UPD in our cohort was approximately 1 in 55 births which is higher than previously reported (King D. A. et al., 2014; Nakka et al., 2019; Yauy et al., 2020; Scuffins et al., 2021). It should be noted that we did not screen for mosaic uniparental disomy events, which could yet increase the incidence of UPD further. Whole chromosome UPiD is the rarest type of UPD encountered in the general population (1 in ∼7,500) compared to UPhD (1 in ∼5,500) and partial UPiD (1 in ∼5,500) (Nakka et al., 2019). However, in our cohort of 164 probands, it was the only type of UPD identified. Whole chromosome isodisomy may present a greater clinical risk, as the probability of unmasking rare and damaging recessive variants increases in proportion to the quantity of DNA affected. It is therefore not surprising that an undiagnosed cohort could have a higher proportion of complete isodisomy events.

Truncating genetic variants in patients with CFSMR are hypothesized to abrogate the normal protein function of TMCO1. All pathogenic variants identified to date are consistent with this hypothesis (Xin et al., 2010; Caglayan et al., 2013; Tender and Ferreira, 2018; Sharkia et al., 2019). TMCO1 is an important regulator of many cellular processes including transcription, cell death, and proliferation, acting on the maintenance of endoplasmic reticulum (ER) Ca^2+^ homeostasis by preventing overfilling of ER stores with Ca^2+^ ions (Wang et al., 2016). This is the first report of *TMCO1* and UPD, an ever-growing list of disorders associated with UPD (King J. E. et al., 2014).

The homozygous variant p. (Pro222Leu) in P2 was first identified in six individuals from two consanguineous families (Camargos et al., 2008). Dystonia 16 (MIM: 612067) is a generalized dystonia, with predominant oromandibular, craniofacial and axial involvement. PACT, the protein counterpart of *PRKRA*, activates PKR, a regulator of how cells respond to viral infections, ER stress, growth factor deprivation, and oxidative stress. The p. (Pro222Leu) variant produces an altered kinetic response to ER-stress. It is thought to foster stronger homo-dimer interactions with itself and with the RNA-activated protein kinase, PKR, leading to an aberrant stress response with a concomitant increase in apoptosis (Vaughn et al., 2015; Burnett et al., 2020). While the age-of-onset for P2 (3 years) is slightly younger than previous reports (average 8 years (range: 4–14 years) (Lange et al., 2021), the phenotype is otherwise typical of Dystonia 16, with predominant dysarthria. The encephalopathic illness (Supplementary Data) may have precipitated earlier onset of presentation (Masnada et al., 2021).

Attributing homozygosity to an inheritance error rather than parental carriers has important implications for family planning and cascade testing. The identification of a homozygous variant would often imply a recurrence risk of 25%. Establishing the source of homozygosity facilitates accurate genetic counselling (recurrence risk is negligible) and avoids the incorrect presumption of non-paternity (King J. E. et al., 2014).

The UPiD event in P3 may represent a benign and incidental finding with no pathological impact. It may equally represent a clinically significant event, but at this time no candidate gene was identified. As the curation of novel disease-gene associations is a continuing effort we stress the importance of periodic reanalysis for P3 (Costain et al., 2018; James et al., 2020).

In summary, our data supports UPD screening as a method of clinical value in the analysis of rare disorders. The incidence of UPiD in our cohort was approximately 1 in 55 births which is higher than previously reported incidences in the general population (Nakka et al., 2019) This may represent an overestimation, or it could suggest that isodisomic events present greater clinical risk. While isodisomy in many instances is benign, the coincidence of isodisomy and a severe congenital disorder should be investigated as potentially causal.

## Data Availability

The datasets presented in this article are not readily available because they relate to paediatric rare disease patients under the care of Children’s Health Ireland. Requests to access the datasets should be directed to Children’s Health Ireland.
