# Supplementary material for: Uniparental disomy screen of Irish rare disorder cohort unmasks homozygous variants of clinical significance in the TMCO1 and PRKRA genes
Source: Front Genet. 2022 Sep 14;13:945296. doi: 10.3389/fgene.2022.945296 (PMC9515794; doi:10.3389/fgene.2022.945296)
Supplement: Supplementary file 1 [file DataSheet1.docx]

**Supplementary Data**

**
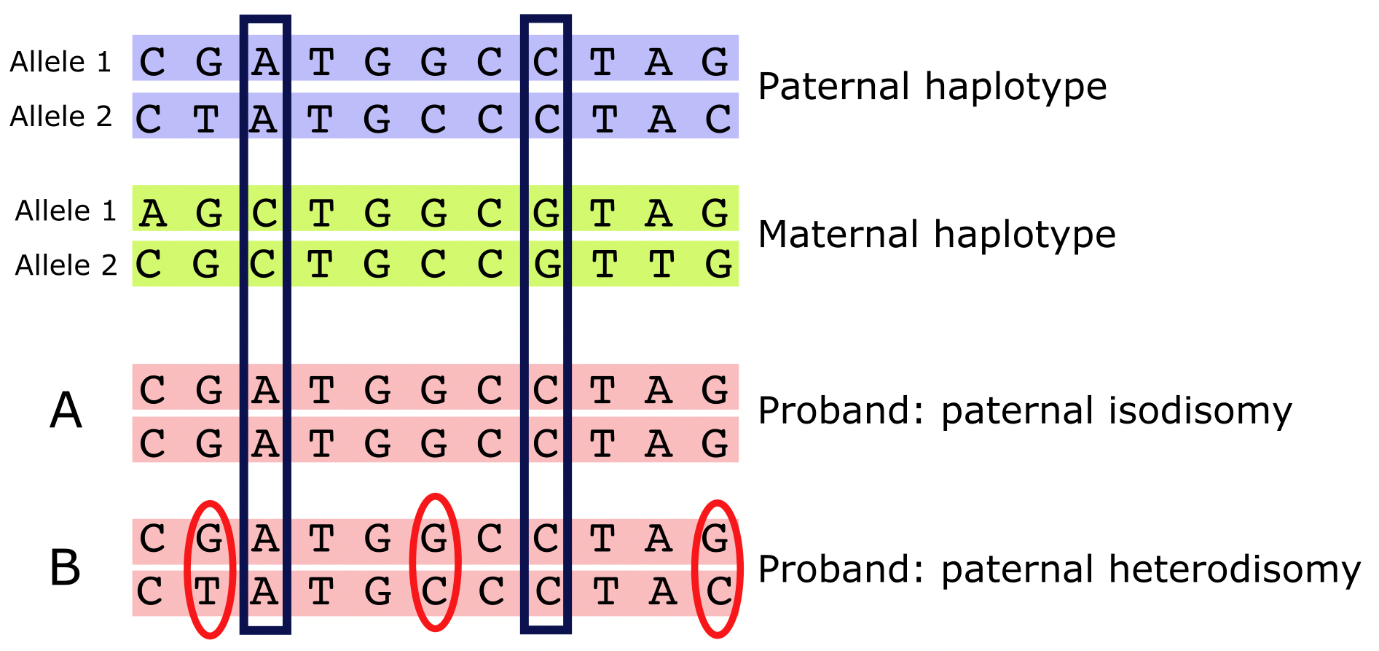
**

**Fig. S1. Overview of UPD assessment method.** For each trio, positions in the genome which were homozygous for different alleles in the parents were examined (shown in boxes above). We examined each chromosome for long regions of Mendelian error in the child (i.e. positions where the child was not heterozygous for the parental alleles). This pattern could be consistent with a uniparental disomy event when there is no loss of coverage in the region. To distinguish between isodisomy and heterodisomy we assessed heterozygosity across the region of Mendelian error. In (A), the proband has inherited two copies of allele 1 from their father in a paternal isodisomic event resulting in a loss of heterozygosity in the region. In (B), the proband has inherited both allele 1 and allele 2 from their father in a paternal heterodisomic event, resulting in no loss of heterozygosity (heterozygous positions highlighted in red).

**1. Clinical summaries**

**1.1 P1**

P1, born to healthy parents, presented at birth with predominantly axial hypotonia. There was no respiratory or feeding difficulties. There were dysmorphic features including triangular-shaped mouth, excessive nuchal skin, and broad, adducted thumbs. The only abnormalities identified on investigations were a hemivertebra identified on chest X-ray, and a bicuspid aortic valve on echocardiography. Magnetic resonance imaging (MRI) of brain and spine, abdominal ultrasound, muscle biopsy, creatine kinase, karyotype, FISH probe for 22q11 deletion, CGH microarray screen and metabolic investigations were all negative.

At follow-up, there was persisting hypotonia, delayed language, gross and fine motor milestones, scoliosis due to her hemivertebra and Sprengel’s deformity of her shoulder. At last review, there was moderate intellectual disability and mild tremor with normal ambulation, and no evidence of ataxia.

**1.2 P2**

P2 was born to healthy parents and had normal developmental milestones until presentation acutely following an intercurrent illness during early childhood where he was sleepy and became unsteady. A diagnosis of viral encephalopathy with ataxia was considered and antiviral treatment was commenced; however, an infectious cause was not identified, even after cerebrospinal fluid (CSF) examination. Initial MRI brain revealed bilateral basal ganglia changes suggestive of a mitochondrial disorder but CSF studies including lactate and MR spectroscopy were normal. There were no seizures and EEG was normal. Muscle biopsy showed normal histology and respiratory chain enzymology. Long-range PCR studies on blood-derived DNA suggested the possibility of multiple mitochondrial DNA deletions, but further extensive genetic testing, including mitochondrial DNA sequencing, was negative. There was incomplete recovery with ongoing dysarthria, ataxia, hypotonia, hyperreflexia, dyspraxia and fatigue. Neurological, infectious disease, rheumatology, and genetic investigations (karyotype and array-CGH) were negative as were cardiology and ophthalmology assessments. Extensive metabolic investigations were also non-diagnostic, including intermediary metabolism/mitochondrial, lysosomal, peroxisomal and glycosylation studies, as well as enzyme studies to exclude glutaric aciduria type 1.

There was no response to a trial of mitochondrial supplements, including, e.g., co-enzyme Q10, carnitine, thiamine, riboflavin and biotin. Repeat neuroimaging showed some volume loss of both caudate nuclei and putamina. P2 requires on-going substantial multi-disciplinary team input, e.g. speech and language therapy, physiotherapy, dietetics and psychology.

- 1. **P3**

P3 was born to healthy parents and presented during early childhood with chronic otitis media. P3 developed a chronic cough and was hospitalized on three occasions with lower respiratory tract infections. Investigations demonstrated T-cell lymphopenia with a relative reduction in both the proportion of CD4+ T_helper_ cells and the proportion of naïve T-cells. Measurement of serum immunoglobulins demonstrated persistently low serum IgM levels. Serum IgG levels were normal, but P3 also demonstrated dysgammaglobulinemia, with persistently poor vaccine specific antibody titres post-vaccination against pneumococcus and *Haemophilus influenzae* type B. His clinical condition improved significantly once he was commenced on immunoglobulin replacement therapy with a reduction in infection burden.
